# Up-regulation of galectin-9 induces cell migration in human dendritic cells infected with dengue virus

**DOI:** 10.1111/jcmm.12500

**Published:** 2015-03-06

**Authors:** Yu-Lin Hsu, Mei-Yi Wang, Ling-Jun Ho, Chuan-Yueh Huang, Jenn-Haung Lai

**Affiliations:** a Graduate Institute of Medical Science, National Defense Medical CenterTaipei, Taiwan; b Graduate Institute of Microbiology and Immunology, National Defense Medical CenterTaipei, Taiwan; c Institute of Cellular and System Medicine, National Health Research InstituteZhunan, Taiwan; d Division of Allergy, Immunology, and Rheumatology, Department of Internal Medicine, Chang Gung Memorial Hospital, Chang Gung UniversityTao-Yuan, Taiwan

**Keywords:** dengue virus, dendritic cells, galectin-9, cell migration, immune response, mitogen-activated protein kinase

## Abstract

Galectin-9 (Gal-9) exerts immunosuppressive effects by inducing apoptosis in T cells that produce interferon-γ and interleukin (IL)-17. However, Gal-9 can be pro-inflammatory in lipopolysaccharide-stimulated monocytes. Using microarray analysis, we observed that Gal-9 was up-regulated in human dendritic cells (DCs) after dengue virus (DV) infection. The investigation into the immunomodulatory effects and mechanisms of Gal-9 in DCs exposed to DV revealed that DV infection specifically increased mRNA and protein levels of Gal-9 but not those of Gal-1 or Gal-3. Blocking p38, but not c-Jun N-terminal kinase or extracellular signal-regulated kinase (ERK), inhibited DV-induced expression of Gal-9. Reduction in Gal-9 by small interference RNA treatment suppressed DV-stimulated migration of DCs towards the chemoattractants CCL19 and CCL21. In addition, DV-induced IL-12p40 production was reduced after knockdown of Gal-9 in DCs. Furthermore, Gal-9 deficiency suppressed DV-induced activation of nuclear factor-κB. Inhibition of DV-induced DC migration under conditions of Gal-9 deficiency was mediated through suppressing ERK activation but not by regulating the expression of CCR7, the receptor for CCL19 and CCL21. Both the reduction in IL-12 production and the suppression of ERK activity might account for the inhibition of DV-induced DC migration after knockdown of Gal-9. In summary, this study reveals the roles of Gal-9 in DV-induced migration of DCs. The findings indicate that Gal-9 might be a therapeutic target for preventing immunopathogenesis induced by DV infection.

## Introduction

The galectins are a subgroup of lectins that bind β-galactoside–containing glycans and have a conserved amino-acid sequence in their carbohydrate recognition domain 1. They are constitutively expressed in a variety of non-immune and immune effector cells, including endothelial cells, macrophages, dendritic cells (DCs) and T and B lymphocytes 2,3. Expression of galectins is also regulated by disease status. Galectin-1 (Gal-1) expression is higher in HIV-1-infected CD4^+^ T cells than in non-infected CD4^+^ T cells and untreated cells 4. Patients with hepatitis B or hepatitis C virus infection have higher levels of Gal-9 in plasma than do healthy controls 5,6. Expression of Gal-9 in synovial tissues of patients with rheumatoid arthritis and in a collagen-induced arthritis model positively correlates with apoptosis of synovial fibroblasts 7. Functionally, binding of galectins to their ligands was shown to mediate cell–cell and cell–pathogen interactions 8.

The interaction between Gal-9 and its ligand, T cell immunoglobulin-3 (Tim-3), on T helper 1 (Th1) cells inhibits T-cell expansion and induces cell death and immune tolerance 9–11. In addition, Gal-9 inhibits pathogenic interleukin (IL)-17-producing effector Th cells and augments immunosuppressive regulatory T cells 12. In addition to its regulatory roles in T cells, Gal-9 expands populations of immunosuppressive macrophages 13, monocytic myeloid-derived suppressor cells 14 and plasmacytoid DC-like macrophages 15. When Gal-9 is deficient, there is greater production of interferon (IFN)-γ by natural killer cells and increased degranulation, which suggests that Gal-9 also behaves as a negative regulator of natural killer cell function 16. By regulating T-cell function and inducing plasma cell apoptosis, Gal-9 attenuates disease severity in MRL/lpr lupus-prone mice 17. The evidence suggests that Gal-9 is critical in mediating immunosuppressive function in various immune conditions.

Gal-9 also functions as a positive pro-inflammatory regulator. According to Matsuura *et al*., Gal-9 in response to lipopolysaccharide stimulation rapidly translocates from the cytosol into the nucleus, interacts with nuclear factor (NF)-IL-6 and induces production of several pro-inflammatory cytokines, including IL-1α, IL-1β and IFN-γ in monocytes 18. Binding of Tim-3 on CD4^+^ Th1 cells to Gal-9 on macrophages also promotes clearance of intracellular pathogens 19. Moreover, Gal-9 expression can be up-regulated and Gal-9 can be released in response to stimulation by pro-inflammatory stimuli 20. Conversely, Gal-9 can induce production of pro-inflammatory cytokines from Th cells through Tim-3-independent mechanisms 21.

Dengue virus (DV) infection is a major public health issue worldwide. Being the most efficient antigen-presenting cells, DCs are the major targeted host for DV 22. We and other researchers previously identified several genes that are either up- or down-regulated by DV infection in human DCs (data not shown) and umbilical endothelial cells 23, of which Gal-9 is one. Importantly, elevation of plasma Gal-9 closely correlates with development of severe haematological manifestations in patients infected with DV 24. To date, no studies have examined the function and role of Gal-9 in DV infection, and it is not known whether Gal-9 has a role in DCs infected by DV. In this report, we demonstrated that DV infection caused up-regulation of Gal-9 expression, thereby inducing DC migration, an important process in DV-mediated pathogenesis. We also described the molecular mechanisms involved in these processes.

## Materials and methods

### Culture medium and reagents

The culture medium consisted of RPMI 1640 (Gibco-BRL, Life Technologies Corporation, Carlsbad, CA, USA) supplemented with 10% foetal bovine serum (FBS; Hyclone, Thermo Fisher Scientific Inc, Waltham, MA, USA). Recombinant granulocyte-macrophage colony-stimulating factor (GM-CSF) and IL-4 were purchased from R&D Systems (Minneapolis, MN, USA). Mitogen-activated protein kinase (MAPK) inhibitors (SB203580 for p38, PD98059 for ERK and SP600125 for JNK) were purchased from Calbiochem (Merck KGaA, Darmstadt, Germany). The antibodies (Abs) recognizing CD80, CD86, CD83 and HLA-DR were purchased from BD Pharmingen (BD Biosciences, Franklin Lakes, NJ, USA). Antibodies recognizing total or phosphorylated c-Jun N-terminal kinase (JNK), p38 and extracellular signal-regulated kinase (ERK) were purchased from Santa Cruz (Santa Cruz Biotechnology, Santa Cruz, CA, USA) or Cell Signaling (Beverly, MA, USA). Antibodies against p65, p50, p52, RelB, c-Rel, Na^+^/K^+^-ATPase α, Toll-like receptor-3 (TLR-3) and upstream stimulatory factor (USF)-2 were purchased from Santa Cruz. Anti-CCR7, anti-IL-12p40 and anti-Gal-9 Abs and chemoattractants, including CCL19 and CCL21, were purchased from R & D. Anti-Tim-3 neutralizing Abs (LEAF™ purified anti-human Tim-3 antibody) were purchased from BioLegend (San Diego, CA, USA).

### Preparation of DCs

Dendritic cells were prepared from purified CD14^+^ monocytes, as previously described 25. In brief, buffy coat (equivalent to 500 ml of whole blood for each) from a blood bank (Taipei, Taiwan) was mixed with Ficoll-Hypaque and, after centrifugation, the layer of peripheral blood mononuclear cells was collected and the cells were incubated with anti-CD14 microbeads at 4–8°C for 15 min. After washing, CD14^+^ cells were isolated using a magnetic-activated cell sorting cell isolation column (Miltenyi Biotech, Bergisch Gladbach, Germany). The purified monocytes were cultured in medium containing 800 U/ml GM-CSF and 500 U/ml IL-4 at a cell density of 1 × 10^6^ cells/ml. The culture medium was changed every other day with fresh medium containing GM-CSF and IL-4, and cells with purity higher than 92% after 5–7 days of culture were used in the experiments 25.

### DV preparation and infection

Preparation of DV has been previously described 25. In brief, DV2 strains, New Guinea C (NGC, ATCC), 16681 and wild-type local Taiwanese strain PL046, were propagated in C6/36 mosquito cells in RPMI containing 5% heat-inactivated FBS and maintained at 28°C for 7 days. Preparation of mock was done using the same procedures, except that buffered saline was substituted for the virus inoculation. The virus titres in supernatants were determined by plaque-forming assays and the viral stocks were stored at −70°C until use 25. Unless otherwise specified, DCs (1 × 10^6^/ml in culture medium) were infected with mock or DV at a multiplicity of infection (MOI) of 5 for 2 hrs at 37°C 25. After viral adsorption, cells were washed and cultured with culture medium in the presence of exogenously added cytokines.

### Determination of virus titre

Determination of virus titre was done according to methods described in our previous report 22. Various dilutions of virus were added to 80% confluent baby hamster kidney-21 cells and incubated at 37°C for 2 hrs. After adsorption, the cells were overlaid with 3 ml of RPMI 1640 containing 1% low-melting-temperature agarose (SeaPlaque; FMC BioProducts, Philadelphia, PA, USA), 1% penicillin, 1% streptomycin and 2% FBS. The cells were incubated for 7 days, fixed with 2% formaldehyde and stained with 0.5% crystal violet. The numbers of plaques were counted, and the results were recorded as plaque forming units per millilitre.

### Quantitative RT/PCR (qRT-PCR)

Total RNA from treated cells was isolated with TRIZOL® reagent (Invitrogen, Carlsbad, CA, USA) as described in our previous report 26. RNA concentrations were measured using Nanodrop (ND 1000 V.3.1.0). Reverse transcription of purified RNA was performed with a random primer (Applied Biosystems, Life Technologies Corporation). The cDNA was prepared for further analysis with quantitative real-time PCR, under the aid of a fluorescent LightCycler® 480 SYBR Green I Master (Roche Diagnostics Corp., Indianapolis, IN, USA) and analysed by the LightCycler® 480 System (Roche). All values were normalized to the level of GAPDH mRNA. All assays were performed in triplicate and repeated in three independent experiments. The primers used were as follows: Gal-9, sense (5′-CTTTCATCACCACCATTCTG-3′), antisense (5′-ATGTGGAACCTCTGAGCACTG-3′); Gal-1, sense (5′-CCTGGACTCAATCATGGCTTGT-3′), antisense (5′-AAGTGCAGGCACAGGTTGTTG-3′); Gal-3, sense (5′-ACAATTCTGGGCACGGTGAA-3′), antisense (5′-TCCCCAGTTATTATCCAGCTTTG-3′); Tim-3, sense (5′-TCCAAGGATGCTTACCACCAG-3′), antisense (5′-GCCAATGTGGATATTTGTGTTAGATT-3′); PDI, sense (5′-GGTGCTGCGGAAAAGCAAC-3′), antisense (5′-ACCTGATCTCGGAACCTTCTG-3′); CD44, sense (5′-GCAACTGAGACAGCAACCAAG-3′), antisense (5′-GCCATTTGTGTTGTTGTGTGAA-3′); TLR-3, sense (5′-TTGCCTTGTATCTACTTTTGGGG-3′), antisense (5′-TCAACACTGTTATGTTTGTGGGT-3′); GAPDH, sense (5′-AGGTGAAGGTCGGAGTCAAC-3′), antisense (5′-CCATGTAGTTGAGGTCAATGAAGG-3′).

### Flow cytometry

The method for determining expression of CD80, CD86, CD83, HLA-DR and CCR7 has been previously described 25,27. Human DCs were collected and washed twice with cold PBS and then stained with PE-conjugated mAbs to CD80 or HLA-DR or FITC-conjugated mAbs recognizing CD86 or CD83 at 4°C for 1 hr. The cells were then analysed and quantified using flow cytometry. The isotype-matched controls were purified mouse IgG1 (BD Pharmingen™). For determination of CCR7 expression, cells were washed twice with cold PBS and incubated with CCR7 antibody at 4°C for 30 min. After washing, biotin-attached antimouse IgG/IgM antibodies were added and incubated for another 30 min. Finally, cells were washed twice with cold PBS and stained with streptavidin-PE at 4°C for 30 min. for flow cytometry analysis. Data were processed and analysed with CellQuest software (BD Biosciences).

### Nuclear extract preparation

Nuclear extracts were prepared as previously described 25. Briefly, the treated cells were left at 4°C in 1 ml of buffer A (10 mM HEPES, pH 7.9, 10 mM KCl, 1.5 mM MgCl_2_, 1 mM DTT, 1 mM PMSF and 3.3 μg/ml aprotinin) for 1 hr, with occasional gentle vortexing. Swollen cells were centrifuged at 17,530 × g. for 3 min. After removal of the supernatants (cytoplasmic extracts), the pelleted nuclei were washed with 300 μl buffer A, and cell pellets were then resuspended in 30 μl buffer C (20 mM HEPES, pH 7.9, 420 mM NaCl, 1.5 mM MgCl_2_, 0.2 mM ethylenediaminetetraacetic acid (EDTA), 25% glycerol, 1 mM DTT, 0.5 mM PMSF and 3.3 μg/ml aprotinin) and incubated at 4°C for 2 hrs with occasional vigorous vortexing. The mixtures were centrifuged at 17,530 × g for 20 min., and the supernatants were used as nuclear extracts.

### Electrophoretic mobility shift assay

Electrophoretic mobility shift assay (EMSA) was performed as previously described 25. Oligonucleotides containing NF-κB binding site were purchased and used as DNA probes (Promega, Madison, WI, USA). The probes were radiolabeled with [γ-32p]ATP using T4 kinase (Promega). For the binding reaction, the radiolabeled NF-κB probe was incubated with 5 μg of nuclear extracts. The binding buffer contained 10 mM Tris-HCl (pH 7.5), 50 mM NaCl, 0.5 mM EDTA, 1 mM DTT, 1 mM MgCl_2_, 4% glycerol and 2 μg poly(dI-dC). The binding reaction proceeded for 20 min. at room temperature. Whenever competition assays were performed, 100-fold molar excess of non-radiolabeled, competitive oligonucleotides (wild-type or mutant) were added, and they were pre-incubated with nuclear extracts for 30 min. before adding radiolabeled probes.

### Western blotting

Enhanced chemiluminescence Western blotting (Amersham, GE Healthcare Life Science, Uppsala, Sweden) was performed as previously described 25. Briefly, equal amounts of proteins were analysed on 10% SDS-PAGE and transferred to a nitrocellulose filter. For immunoblotting, the nitrocellulose filter was incubated with TBS-T containing 5% nonfat milk for 1 hr and then blotted with antisera against individual proteins overnight. After washing with TBS-T, the filter was incubated with secondary Ab conjugated to horseradish peroxidase for 1 hr. The filter was then incubated with the substrate and exposed to an x-ray film. After scanning, comparing the intensity of bands on Western blots or EMSA was done using an alpha digidoc1201 software.

### Chemotaxis assay

Chemotaxis assays were performed according to our previous report 27. In brief, DCs treated under the different conditions migrated through a polycarbonate filter (pore size 5 μm) in 24-well transwell chambers (Corning Costar, Corning Incorporated Life Sciences, Tewksbury, MA, USA). The lower chamber of the transwell cassette contained serum-free RPMI containing 600 μl of 0.1% BSA with or without 100 ng/ml CCL19 or CCL21 (R&D Systems). DCs (1 × 10^5^) in 100 μl of serum-free medium containing 0.1% BSA were loaded in the upper chamber and incubated for 3–5 hrs at 37°C. Then, cells migrating from the upper chamber to the lower chamber were counted by flow cytometry. CellQuest software was used to determine the acquired events during a fixed time period of 60 sec. in a FACScan.

### Knockdown of Gal-9 or TLR-3 by siRNA silencing

For knockdown experiments, all small interfering RNAs (siRNAs; Stealth RNAi™ siRNA, Invitrogen, Life Technologies Corporation) were transfected by electroporation using a BTX cuvette (Harvard Apparatus, Inc., Holliston, MA, USA) according to the manufacturer's instructions. The siRNA sequences used were as follows. Gal-9-1: UGAGGUGGAAGGCGAUGUGGUUCCC; Gal-9-2: UGUUGUGGACCACAGCGUUCUCAUC; TLR-3-1: GCAAACCCUGGUGGUCCCAUUUAUU; TLR-3-2: CCUGAGCUGUCAAGCCACUACCUUU; TLR-3-3: CCACCACCAGCAAUACAACUUUCUU. Cells were collected and resuspended in modified Eagle's Minimum Essential Medium (Opti-MEM, Invitrogen, Life Technologies Corporation) containing 300 nM siRNA. After transfer to the cuvette, the cells were electroporated with one pulse at 300 V for 3 msec. To determine the efficiency of protein knockdown, at 48 hrs post-transfection, cells were lysed in RIPA buffer and immunoblotted with the indicated Abs.

### Determination of cytokines and chemokines by ELISA

Standard ELISA methods were used to measure concentrations of Gal-9 (R&D Systems), cytokines and chemokines such as IL-12p40, IL-12p70, MIP-1β (macrophage inflammatory protein [MIP]), monocyte chemoattractant protein 2 (MCP-2), tumour necrosis factor-alpha (TNF-α), IL-6, transforming growth factor-beta1 (TGF-β1), IL-18, IL-23, IL-27, and IL-1β (R&D Systems) and IFN-α (Verikine™ Human IFNα ELISA Kit, PBL Assay Science, Piscataway, NJ, USA).

### Statistical analysis

The results were expressed as the mean ± SD of triplicate experiments. Statistical comparisons were performed with one-way anova. When anova showed significant differences between groups, Bonferroni's *post hoc* test was used to determine the specific pairs of groups that significantly differed. A *P* < 0.05 was considered to indicate statistical significance. Asterisks indicate values that are significantly different from control (*P < 0.05; **P < 0.01; ***P < 0.001).

## Results

### DV infection-induced Gal-9 expression

**Figure 1:**
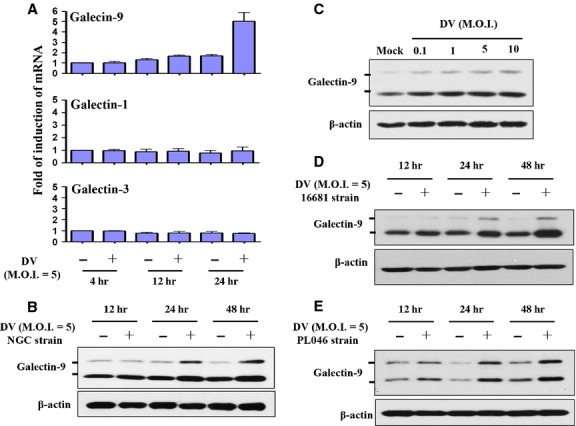
Dengue virus (DV) infection activated Gal-9 in human DCs. Human DCs (1 × 10^6^ cells/ml) were infected by mock or DV NGC strain at an MOI of 5. Quantitative RT-PCR was used to determine expression of MRNAs of the Gal-9, Gal-1 and Gal-3 genes (A), and Gal-9 protein levels were determined by Western blotting (B). Cells were infected by mock or DV (NGC strain) at various MOIs for 48 hrs, and Gal-9 expression was determined by Western blotting (C). Similarly, protein levels of Gal-9 in human DCs infected by different DV2 strain 16681 (D) or PL046 (E) at an MOI of 5 for various time-points were determined. The data shown are from three independent experiments examining different donor DCs.

To confirm the results in microarray analysis (data not shown), we applied quantitative RT/PCR assays and showed that DV infection-induced expression of Gal-9 but not Gal-1 or Gal-3 mRNA (Fig.1A). This suggests that DV infection selectively activated Gal-9 but not other galectin signals in DCs. Western blots confirmed that the Gal-9 protein was up-regulated in DCs infected by DV (Fig.1B). Importantly, the dose-relationship evaluation showed a significant induction of Gal-9 expression in DCs infected by DV at an MOI of 0.1-1 (Fig.1C). When DCs were infected by two other DV2 strains, 16681 and PL046, Gal-9 expression was consistently observed (Fig.1D and E). Since both Gal-9 and Tim-3 can be expressed on DCs 28, we examined whether DV infection regulated expression of Tim-3 and other potential receptors of Gal-9. As shown in Figure S1, DV infection did not induce expression of Gal-9 receptors, including Tim-3, protein disulphide isomerase (PDI) or CD44 on DCs. It has been reported that DV infection in human cancer cell lines is likely dependent on TLR-3 that mediates the subsequent secretion of IFN-α/β and viral replication 29, we thus introduced siRNAs of TLR-3 into cells to knockdown this molecule. The analysis on mRNA expression revealed that DV infection-induced TLR-3 expression and effective knockdown of TLR-3 had no effect on DV-induced Gal-9 mRNA and protein expression (Fig. S2A and B). As controls, knockdown of TLR-3 reduced Poly I:C-induced Gal-9 expression by Western blotting (Fig. S2C).

### DV induced Gal-9 expression in a p38-dependent manner

**Figure 2:**
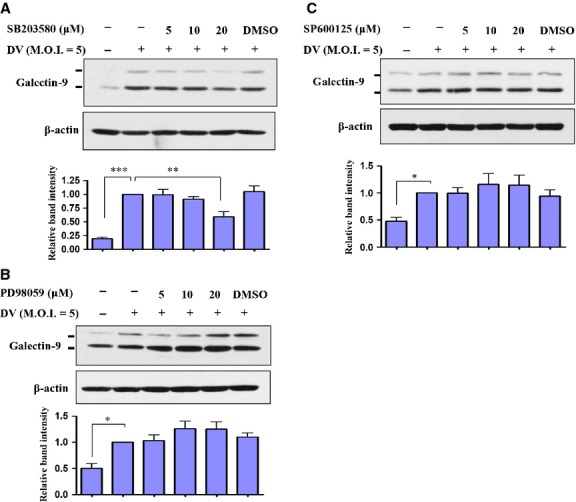
DV-induced Gal-9 expression was p38-dependent. Human DCs were pre-treated with a p38 inhibitor (SB203580), ERK inhibitor (PD98059) or JNK inhibitor (SP600125) for 2 hrs at various concentrations and then infected by mock or DV (NGC strain) for 48 hrs. Total cell lysates were collected, and expression of Gal-9 and β-actin was determined by Western blotting (A–C). The data show representative results and analysis pooled from at least three independent experiments examining different donor DCs. The analysis was performed by anova as described in the Materials and methods. *P < 0.05, **P < 0.01, ***P < 0.001.

Mitogen-activated protein kinases are critical in Gal-9 expression induced by various stimuli. Therefore, we examined whether MAPKs also have roles in DV-induced Gal-9 expression. Cultured DCs were pre-treated with various doses of a p38 inhibitor (SB203580) for 2 hrs and then infected with DV for an additional 48 hrs, after which Gal-9 expression was determined. As shown in Figure2A, p38 inhibitor treatment reduced DV-induced expression of Gal-9. However, under similar conditions, neither an ERK inhibitor (PD98059, Fig.2B) nor a JNK inhibitor (SP600125, Fig.2C) significantly reduced Gal-9 expression in DV-infected DCs.

### Knockdown of Gal-9 impaired DV-induced DC migration

**Figure 3:**
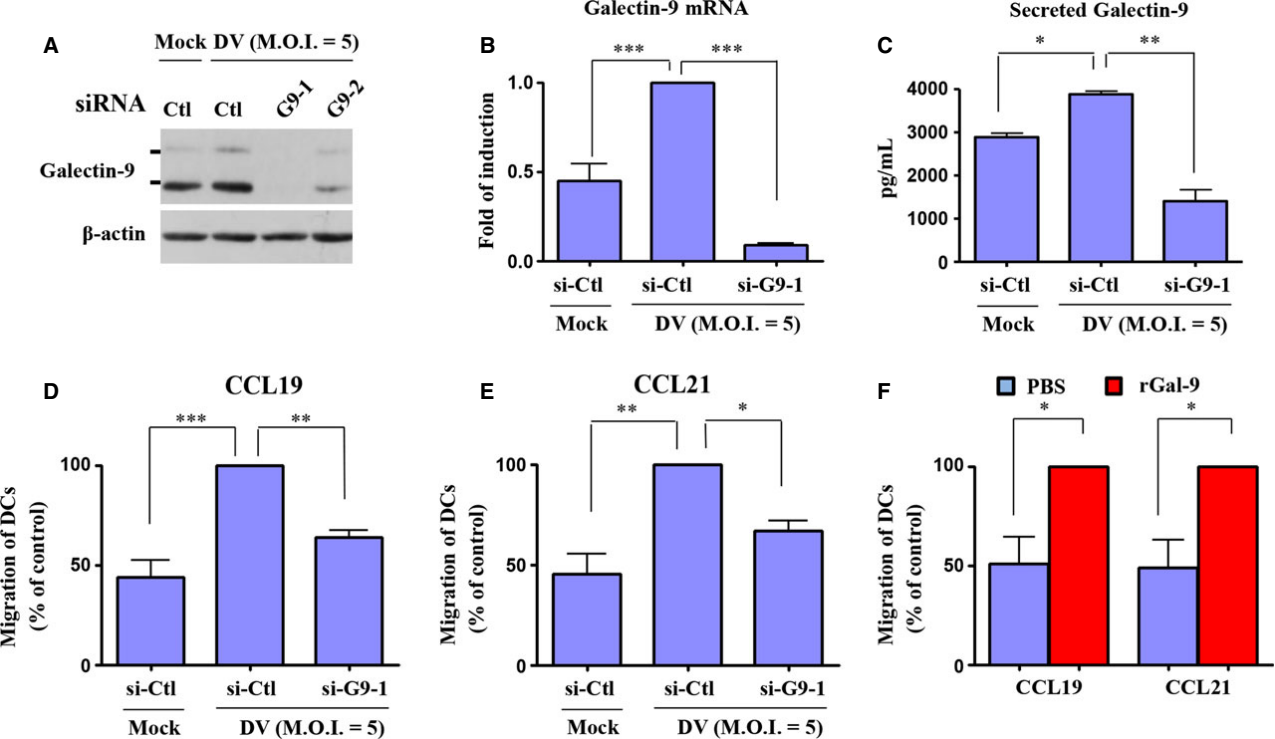
Defective migration of DV-infected DCs deficient in Gal-9. Human DCs were transfected with different siRNAs (si-Ctl, si-G9-1 or si-G9-2) for 24 hrs and then infected with mock or DV for 48 hrs. Expression of Gal-9 protein and Gal-9 mRNA was determined by Western blotting (A) and quantitative RT/PCR (B), respectively. In addition, the supernatants were collected for the determination of Gal-9 concentrations (C). Cells transfected with control siRNA (si-Ctl) or Gal-9 siRNA (si-G9-1) for 24 hrs were infected by mock or DV for additional 48 hrs. Cells were collected for measurement of chemotactic activity by transwell assays using CCL19 (D) or CCL21 (E) as a chemoattractant. In (F), the cells were treated with Gal-9 recombinant protein (rGal-9, 10 μg/ml) or PBS for 48 hrs and then collected for determination of chemotactic activity by transwell assays. DV-infected cells treated with control siRNA transfection (D and E) or rGal-9-treated cells (F) were defined as 100%. The data show results pooled from at least three independent experiments examining different donor DCs. The analysis was performed by anova. *P < 0.05, **P < 0.01, ***P < 0.001. Ctl, control.

Gal-9 has roles in regulating cell adhesion 8. To evaluate this possibility in DV-infected DCs, Gal-9 was knocked down by introducing siRNA into DCs, as described in the Materials and Methods. As shown in Figure3A, two different siRNAs of Gal-9 successfully suppressed Gal-9 expression in DCs. In subsequent studies, siRNA-1 of Gal-9 (si-G9-1) was used for knockdown of Gal-9. Quantitative RT/PCR also demonstrated that DV-induced expression of Gal-9 mRNA decreased in DCs after si-G9-1 treatment (Fig.3B). Furthermore, DV infection in DCs also increased the secretion of Gal-9 into the culture medium and knockdown of Gal-9 effectively reduced the concentration of Gal-9 in the supernatant (Fig.3C). Subsequently, chemotaxis assays were conducted to determine whether knockdown of Gal-9 might affect DV-induced migration of DCs, which was demonstrated in our previous report 27. The results suggest that either CCL19 or CCL21 (an attractant chemokine placed in the lower chamber of the transwell) attracted migration of DV-infected DCs. The effects were greatly reduced in DV-infected DCs that were deficient in Gal-9 (Fig.3D and E). As additional positive controls, we showed that DCs treated with recombinant Gal-9 efficiently migrated towards CCL19 and CCL21 (Fig.3F). Because of the fact that lack of induction of Tim-3 (Fig. S1) does not mean that Tim-3 is not involved in DV-induced migration of DCs, the interaction between Gal-9 and Tim-3 was blocked by addition of anti-Tim-3 neutralizing Abs 30 and migration assay was performed accordingly. The results showed that the neutralizing Abs did recognize Tim-3 on DCs (Fig. S3A) and blocking interaction between Gal-9 and Tim-3 did not affect DV-induced DC migration (Fig. S3B). According to Jayaraman *et al*. that induction of the Gal-9-Tim-3 pathway may inhibit activation of T cells and growth of intracellular pathogens like *Mycobacteria tuberculosis* 19, we investigated whether induction of Gal-9 in DV-infected DCs affects production of DV particles. Plaque assays showed that production of DV in DV-infected DCs was not affected by Gal-9 deficiency (Fig. S4).

### Gal-9 knockdown did not affect expression of maturation and activation markers on DV-infected DCs

After infection by pathogens in the periphery, DCs mature and migrate from the periphery to lymph nodes. We previously demonstrated that DV infection drives DC maturation and induces expression of several activation and maturation markers on the cell surface 22. Because earlier studies also indicated that Gal-9 could induce maturation of DCs 31, we examined whether Gal-9 was necessary for expression of activation and maturation markers on DV-infected DCs. Expression of several cell-surface markers was determined by flow cytometry after DV infection of untreated DCs and DCs deficient in Gal-9. As shown in Figure S5, knockdown of Gal-9 did not affect DV-induced expression of several markers on DCs, including CD80, CD83, CD86 and HLA-DR. These results suggest that Gal-9 is not necessary for DV-induced maturation and increased expression of activation markers on DCs.

### DV infection reduced IL-12p40 production in DCs

**Figure 4:**
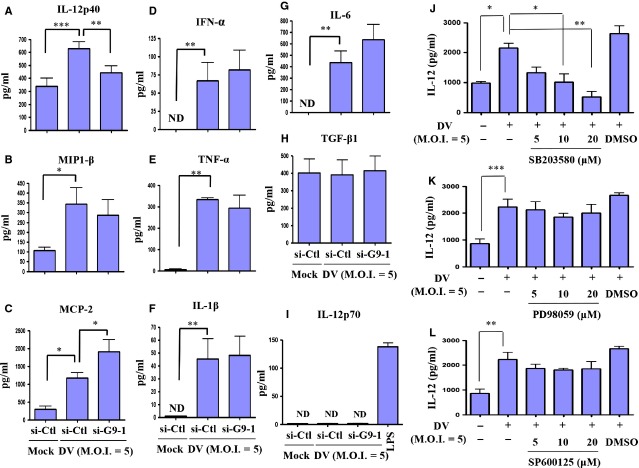
DV infection-induced production of IL-12 was Gal-9- and p38-dependent. Human DCs were transfected with control siRNA (si-Ctl) or Gal-9 siRNA (si-G9-1) for 24 hrs and were then infected by mock or DV for an additional 48 hrs. The supernatants were collected for determination of levels of IL-12p40 (A), MIP1-β (B), MCP-2 (C), IFN-α (D), TNF-α (E), IL-1β (F), IL-6 (G), TGF-β1 (H) and IL-12p70 (I) by ELISA. In (J–L), before infection by DV, DCs were pretreated with various doses of the p38 inhibitor SB203580 (J), the ERK inhibitor PD98059 (K), or the JNK inhibitor SP600125 (L) for 2 hrs. The data show results pooled from at least three independent experiments. The analysis was performed by anova. *P < 0.05, **P < 0.01, ***P < 0.001. Ctl, control.

The effect of Gal-9-mediated DC migration in DV infection was further analysed to examine factors that might be responsible for this event. Previous studies demonstrated that IL-12p40 may have a role in mediating migration of DCs 32; we thus determined whether Gal-9 deficiency affects IL-12p40 production. In addition, production of several chemokines and cytokines was measured in the analysis. Untreated DCs or si-G9-1-treated DCs were infected with mock or DV for 48 hrs, and the supernatants were collected for measurement of several mediators potentially involved in DV-induced migration of DCs. We were surprised to observe that, among the examined cytokines and chemokines, IL-12p40 was significantly reduced in si-G9-1-treated DCs, as compared with DCs treated with control siRNA (Fig.4A). There was also a modest reduction in MIP-1β; however, the difference was not statistically significant (Fig.4B). In contrast, production of MCP-2 was greater in si-G9-1-treated DCs than in DCs treated with control siRNA (Fig.4C). The significance of increased MCP-2 in DV-infected DCs deficient in Gal-9 is currently unclear. Under the same condition, DV-induced production of IFN-α, TNF-α, IL-1β and IL-6 was not affected by knockdown of Gal-9 (Fig.4D–G). DV infection compared to mock infection did not significantly induce production of TGF-β1 (Fig.4H). In contrast to increased production of IL-12p40 in DV-infected DCs, we observed that DV infection did not induce detectable concentration of IL-12p70 in the culture medium (Fig.4I). Furthermore, the production of IL-18, IL-23 and IL-27 was also under detection limit in DV-infected DCs (data not shown). Subsequently, the mechanisms of DV infection-induced IL-12p40 production were examined. Because p38 was demonstrated to regulate DV-induced Gal-9 expression (Fig.2) and Gal-9 played a role in DV-induced IL-12p40 production, we tested the hypothesis that p38 might regulate DV-induced IL-12p40 production. The results confirmed that inhibition with p38 inhibitor SB203580 (Fig.4J), but neither ERK inhibitor PD98059 (Fig.4K) nor JNK inhibitor SP600125 (Fig.4L), suppressed DV-induced production of IL-12p40.

### Impairment of DV-activated NF-κB signalling in DCs deficient in Gal-9

**Figure 5:**
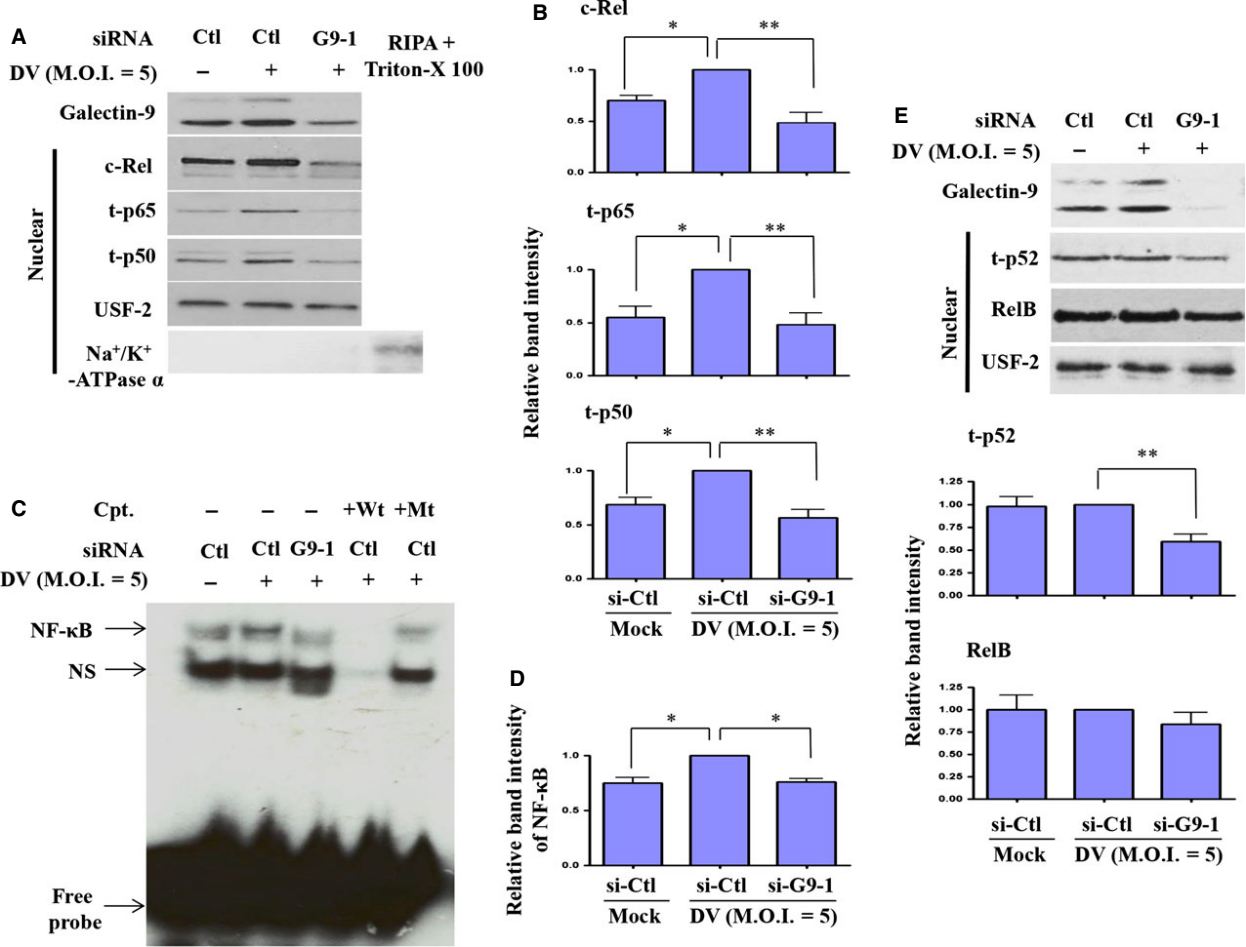
Interference of Gal-9 expression impaired DV-induced activation of NF-κB. Human DCs were transfected with control siRNA (si-Ctl) or Gal-9 siRNA (si-G9-1) for 24 hrs and then infected by mock or DV for an additional 24 hrs. The nuclear extracts were collected for determination of expression of c-Rel, t-p65 (total p65), t-p50 and USF-2 (an internal control) by Western blotting (A). To exclude the possibility of contamination of membrane proteins, the samples were also probed with antibodies against Na^+^/K^+^-ATPase α, a membrane marker. The positive control was the cellular extract, after removing cytoplasmic portion, treated with RIPA buffer in the presence of Triton-X100. Such a preparation contained membrane proteins (A). The densitometry data pooled from three independent experiments using different donor cells were analysed (B). The DNA-binding activity of NF-κB in nuclear extracts was determined by EMSA (C). Densitometry data pooled from three independent experiments using different donor cells were analysed (D). Nuclear extracts pre-incubated with wild-type or mutant κb oligonucleotides served as controls. In (E), similar to those in (A), the levels of both nuclear p52 and RelB were examined. To control the equal amounts of loaded nuclear proteins, the levels of USF-2 protein were used as the backgrounds to normalize individual band intensities shown in EMSA (data not shown). Cpt, competitors; Wt, wild-type; Mt, mutant. The analysis was performed by anova. *P < 0.05, **P < 0.01. Ctl, control.

The selective reduction in IL-12 and, less potently, MIP-1β but not MCP-2 or IFN-α in DV-infected DCs deficient in Gal-9 suggests that transcription factors capable of regulating the former two, but not the latter two mediators are responsible for the observed effects. The NF-κB family proteins are such transcription factors. We previously demonstrated that DV infection effectively activates the NF-κB signalling pathway, and we sought to determine in thisstudy whether knockdown of Gal-9 affects NF-κB activity, a phenomenon that could explain the decrease in IL-12 production. After treatment, nuclear extracts under varying conditions were collected, and the levels of several NF-κB family members were determined by Western blotting. As shown in Figure5A and B, the levels of DV-induced nuclear c-Rel, p65 and p50 were decreased in DCs deficient in Gal-9. EMSA analysis showed that NF-κB DNA-binding activity was greatly reduced in DV-infected DCs previously transfected with si-G9-1, as compared with those transfected with control siRNA (Fig.5C and D). To determine whether the activation of p52/RelB subunits, mediators of the noncanonical NF-κB signalling pathway, also plays roles in DV-induced activation of DCs as well as the effects of Gal-9 deficiency, the nuclear extracts were prepared and analysed. As shown in Figure5E, DV infection did not enhance nuclear levels of p52 and RelB. Interestingly, knockdown of Gal-9 reduced nuclear p52 but not RelB level in DV-infected DCs. Because p52 was not induced after DV infection of DCs, the significance of this protein on Gal-9-mediated DC migration is currently not clear.

### Knockdown of Gal-9 affected CCL19-mediated activation of ERK in DV-infected DCs

**Figure 6:**
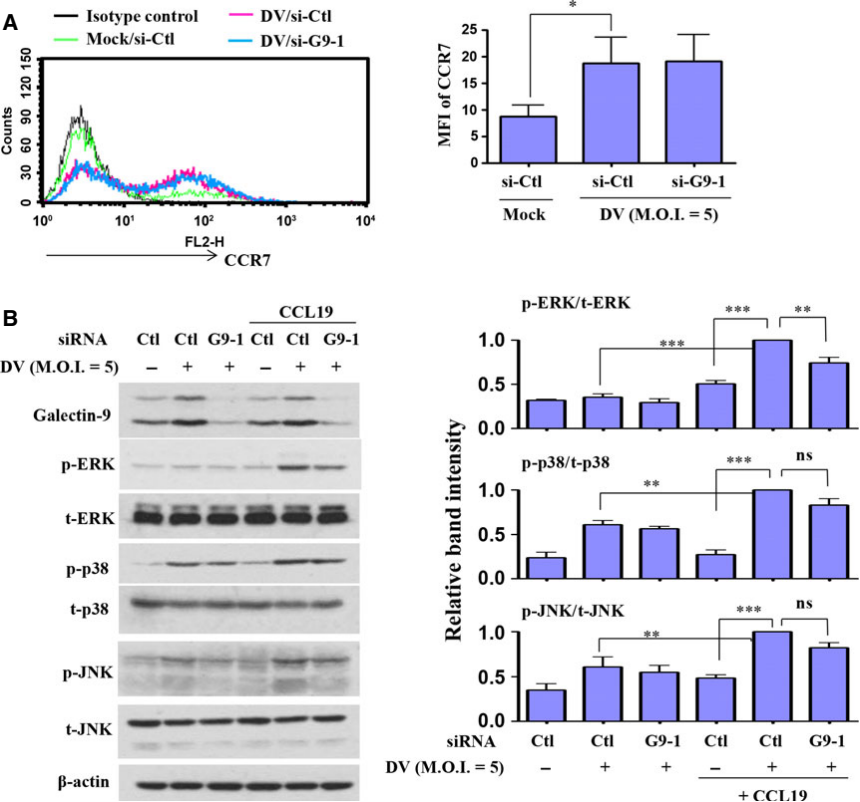
Defective CCL19-mediated activation of ERK in DV-infected DCs with knockdown of Gal-9. Human DCs transfected with control siRNA (si-Ctl) or Gal-9 siRNA (si-G9-1) for 24 hrs were infected by mock or DV for an additional 48 hrs. Cells were collected for measurement of CCR7 expression by flow cytometry (A). Before collection, uninfected DCs and DCs infected with mock or DV were treated with CCL19 200 ng/ml for 2 min. (for determination of p-ERK expression), CCL19 500 ng/ml for 5 min. (for determination of p-p38 expression) or CCL19 500 ng/ml for 30 min. (for determination of p-JNK expression), and the cell lysates were collected individually to determine expression of total and activated MAPKs by Western blotting (B). The results pooled from at least three independent experiments using different donor cells are presented next to the representative figures. The analysis was performed by anova. *P < 0.05, **P < 0.01, ***P < 0.001. Ctl, control; ns, non-significant; MFI, mean fluorescence intensity.

**Figure 7:**
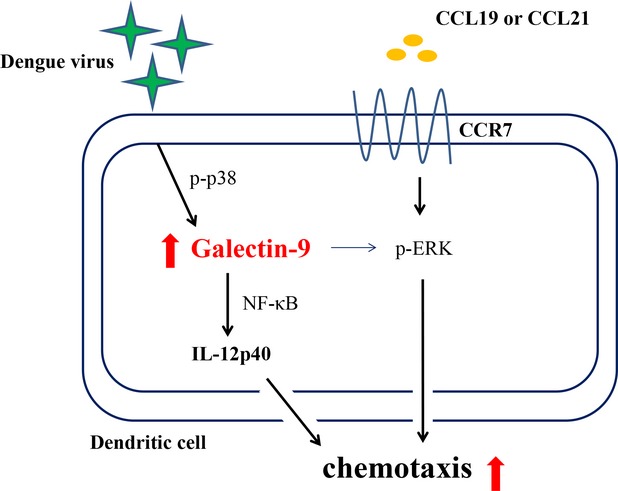
An illustration showing the role of Gal-9 in DV infection-induced chemotaxis in DCs. Infection of DCs by DV induced expression of Gal-9, which was p38-dependent. Gal-9 was responsible for DV-induced IL-12 expression and NF-κB activation. Gal-9 deficiency impaired DV-induced chemotaxis of DCs towards the chemoattractants CCL19 and CCL21; however, expression of CCR7 on DCs was unaffected. The reduced chemotactic effects in DV-stimulated DCs with deficiency in Gal-9 appeared to be because of suppression of ERK activation and IL-12 production.

While siRNA knockdown of Gal-9 impaired DV-induced DC migration towards the chemoattractants CCL19 and CCL21, we anticipated that expression of the CCL19 and CCL21 main receptor, CCR7, would be reduced. Unexpectedly, although infection of DCs by DV up-regulated expression of CCR7, this effect was not affected in flow cytometry analysis of DCs deficient in Gal-9 (Fig.6A). We then wondered whether inhibition of IL-12 production in Gal-9–deficient conditions in DCs by itself was already adequate and enough to explain the reduction in DV-induced cell migration. As shown in Figure S6, the neutralization of IL-12p40 although significantly only modestly suppressed DV-induced DC migration. The results suggest that the factor of IL-12 reduction might not be adequate to explain the effects of Gal-9 deficiency in causing reduced cell migration. We then investigated whether CCR7-mediated intracellular signalling pathways might be impaired under Gal-9-deficient conditions in DCs. DV-infected DCs with or without a deficiency in Gal-9 were treated with CCL19 for various periods of time before cell collection, after which expression of phosphorylated forms of MAPKs was determined by Western blotting. As shown in Figure6B, treatment with CCL19 chemokine did not further increase DV-induced expression of Gal-9; however, CCL19 treatment significantly enhanced DV-induced activation of MAPKs, including ERK, JNK and p38. In DCs deficient in Gal-9, the enhanced activities of MAPKs induced by CCL19 stimulation were attenuated. Most importantly, reduction in the ERK activity was statistically significant. We also noted that both JNK and p38 activities were non-significantly reduced in Gal-9-deficient DCs as compared with control DCs treated with CCL19 in the presence of DV infection. As shown in Figure S7, in a side-by-side comparison, the results showed that knockdown of Gal-9 affected DV-induced DCs migration in the presence or absence of stimulation by the chemoattractants CCL19 and CCL21. These results suggest that both reduced IL-12 production and interference in CCR7-mediated ERK activation together account for the defective migration of DCs deficient in Gal-9 after exposure to DV infection. The mechanism by which Gal-9 might modulate DV-induced DC migration is illustrated in Figure7.

## Discussion

Similar to our findings in DCs, researchers identified Gal-9 as a gene induced in human umbilical vein endothelial cells after DV infection 23. In addition to DV, the induction of Gal-9 can be observed in different host cells infected by different viruses. A recent observation demonstrated the up-regulation of Gal-9 in transplant recipients with reactivated human cytomegalovirus infection, in which IFN-β is likely to play crucial roles in mediating this effect 33. The increase in plasma levels of Gal-9 has also been detected in patients with influenza virus infection compared to those with pneumococcal pneumonia or healthy individuals 34. Furthermore, the increase in plasma concentrations of Gal-9 in HIV-1-infected patients is mainly reflected in monocytes and DCs displaying the highest expression levels and that correlates well with HIV-1 viral loads 35. Moreover, both Tim-3 on virus-specific CD8 T cells and Gal-9 in Kupffer cells, shown to be increased in patients with chronic hepatitis B virus infection, may interact with each other and contribute to the deletion of T cells infiltrating the virus-infected liver 5. High serum levels of Gal-9 have also been demonstrated in patients with hepatitis C virus infection and liver Kupffer cells appear to be crucial cells producing this molecule 36. The detection of markedly elevated plasma Gal-9 levels after DV infection in patients with severe haematological manifestations suggests that this molecule is important in disease pathogenesis 24. In this study, we demonstrated that DV infection specifically induced expression and activation of Gal-9 but not Gal-1 or Gal-3. In addition, induction of Gal-9 was consistently observed in DCs infected by different DV2 strains, including NGC and the PL046 and 16681 strains. More importantly, induction of Gal-9 was detected after infection of DV at low titres of MOIs 0.1–1. By chemical inhibition of kinase activity, we showed that, among MAPKs, only p38 was responsible for DV infection-induced expression of Gal-9 in DCs.

Tim-3 is a major binding receptor for Gal-9 10. In addition, evidence suggests the presence of Tim-3-independent Gal-9 mechanisms 21. Indeed, PDI rather than Tim-3 appears to be crucial for Gal-9 to regulate T-cell function, T-cell migration and HIV infection 37. Furthermore, a study found that Gal-9 administration increases the number of Tim-3^+^ CD86^+^ mature DCs *in vivo* and *in vitro* 38. Interestingly, we observed that three major Gal-9 receptors, namely Tim-3, PDI and CD44, were not induced on DCs after DV infection. Moreover, Gal-9 deficiency did not affect expression of DC maturation and activation markers such as CD80, CD83, CD86 and HLA-DR. Furthermore, blocking the interaction between Tim-3 and Gal-9 through anti-Tim-3 neutralizing Abs did not suppress DV-induced DC migration (Fig. S3). The results suggest that the effects of Gal-9 observed in this study were mediated through an interaction with non-Tim-3 receptors.

The downstream signalling pathways of Gal-9 are largely unknown. We previously reported that DV infection activated the NF-κB signalling pathway in DCs 27. In contrast, the p52/RelB subunits involved in non-canonical NF-κB signalling pathway were not activated in DV infection compared to mock infection of DCs (Fig.5C). In this study, we also demonstrated that Gal-9 knockdown down-regulated DV infection-induced nuclear translocation of c-Rel, p65 and p50, as well as the DNA-binding activity of NF-κB. Interestingly, under the reduced level of Gal-9, the nuclear expression of p52 reduced. The significance of this observation is not clear at this moment. Accompanied by this observation, we were also surprised to find that mainly IL-12, but not several examined cytokines or chemokines, was down-regulated in DV-infected DCs, although NF-κB may also have roles in regulating expression and secretion of some of these cytokines and chemokines. These results suggest that the mechanisms underlying production of chemokines and cytokines in DV-infected DCs are complex. Clearly, additional studies are necessary to resolve this issue.

Gal-9 knockdown resulting in reduction in DV-induced IL-12 production may have a role in the attenuated migration of DCs towards CCL19 and CCL21. It has been shown that, after infection by a *Francisella tularensis* live vaccine strain, DCs secrete IL-12 and migrate towards CCL19 in an IL-12 receptor β1- and homodimeric IL-12p40-dependent manner 39. That study also demonstrated that IL-12 receptor β1 signalling is crucial in post-infection DC migration from the lung to the draining lymph node 39. In another example, Khader *et al*. showed that, in mice deficient in IL-12p40, migration of DCs from the lung to the draining lymph node is defective in response to *M. tuberculosis* infection 32. Importantly, introduction of IL-12p40 homodimer into IL-12p40-deficient DCs restores *M. tuberculosis*-induced DC migration 32. We are surprised to find that neutralizing IL-12p40 although significantly but only modestly suppressed DV-induced migration of DCs. The present results thus support the notion that reduced IL-12 secretion in DV-infected DCs deficient in Gal-9 accounted only in part for decreased DC migration after DV infection. Regarding the rest of the cytokines and chemokines examined, the results showed that most of them were either not induced after DV infection compared to mock infection or far under the detection limit by ELISA. It suggests that the roles of these cytokines or chemokines in DV-induced DC migration may be limited. It has been shown that under hypoxia condition, bone marrow-derived DCs from mice increase migratory capacity, express elevated levels of CD86; however, production of the cytokines such as IL-12p70, IL-10, IL-6, TNF-α, IL-1β and IL-23 is reduced 40. Together with the results in this study, it seems to indicate that IL-12p40, likely a homodimer, may play more important roles than IL-12p70 in mediating DV-induced DC migration.

Jung *et al*. found that sphingosine kinase inhibitor inhibits migration of DCs towards CCL19 through down-regulation of CCR7 and that p38 activity was suppressed 41. While Gal-9 deficiency impaired DV-induced DC migration towards the chemoattractants CCL19 and CCL21, the receptor of these two chemokines, CCR7, was not affected in our study. This suggests that defective migration of Gal-9-deficient cells may be caused by impairment of CCR7-mediated downstream signalling. Humrich *et al*. found that infection of DCs by vaccinia virus caused defective cell migration towards CCL19 and CXCL12 42. The levels of expression of these chemokine receptors were unaffected by infection with the virus. Examination of chemokine receptor-mediated signalling pathways revealed reduced expression of total ERK1, which also results in a reduced level of the active phosphorylated form of ERK1 42. In the present report, we demonstrated that the total amount of ERK was not affected in DV-infected DCs under the deficiency of Gal-9; however, the active phosphorylated form of ERK was significantly inhibited. Meanwhile, although the phosphorylated forms of all MAPKs, including JNK, p38 and ERK, were reduced in Gal-9-deficient cells, only the reduction in phosphorylated ERK was statistically significant. It is thus possible that all MAPKs might contribute to reduced migration of DCs during exposure to DV infection and that, among them, ERK plays the most important roles.

Galectins have very diverse functions. Herpesvirus 1 can adopt Gal-3 as a mediator to enhance viral attachment to human corneal keratinocytes 43. In HIV infection, the virus exploits Gal-1 to enhance interaction of gp120 and CD4 and promote virus attachment and replication 44. Interestingly, a previous study demonstrated that tumour cells with higher Gal-9 expression appear to have reduced metastatic capacity, suggesting that Gal-9 is involved in suppression of tumour cell metastasis 45. This mechanism likely involves inhibition of binding of adhesive molecules on tumour cells to ligands on vascular endothelium and the extracellular matrix 45. It has also been shown that knockdown of Gal-9 (but not Gal-3, -4 or -8) selectively disrupts endolyn polarity and trafficking and affects apical or basolateral cell surface compartmentalization in kidney cells 46,47. In this study, we showed that DV-infected DCs produced Gal-9 that may facilitate their migration towards for example lymphoid organs. The mechanisms likely involved the regulation of IL-12 production and CCR7-mediated activation of MAPKs, especially ERK. Evidently, there is much to learn regarding how the galectin family members work in different tissues and pathological conditions.

## Supporting Information

**Figure S1** Expression of mRNA of Gal-9 receptors on DV-infected DCs. **Figure S2** Knockdown of Toll-like receptor-3 (TLR-3) did not affect DV-induced Gal-9 expression. **Figure S3** Blocking interaction between Tim-3 and Gal-9 did not affect DV-induced DC migration. **Figure S4** DV replication and production in infected DCs was independent of Gal-9. **Figure S5** DV infection-induced expression of activation and maturation markers was not affected in Gal-9-deficient cells. **Figure S6** Neutralizing IL-12 significantly but only modestly suppressed DV-induced DC migration. **Figure S7** Chemotaxis of DV-infected DCs, with or without Gal-9 deficiency, towards the chemoattractants CCL19 and CCL21.
