# Supplementary material for: Up-regulation of galectin-9 induces cell migration in human dendritic cells infected with dengue virus
Source: J Cell Mol Med. 2015 Mar 6;19(5):1065–76. doi: 10.1111/jcmm.12500 (PMC4420608; doi:10.1111/jcmm.12500)
Supplement: Supplementary file 1 [file jcmm0019-1065-sd1.doc]

**Supporting Information**

**
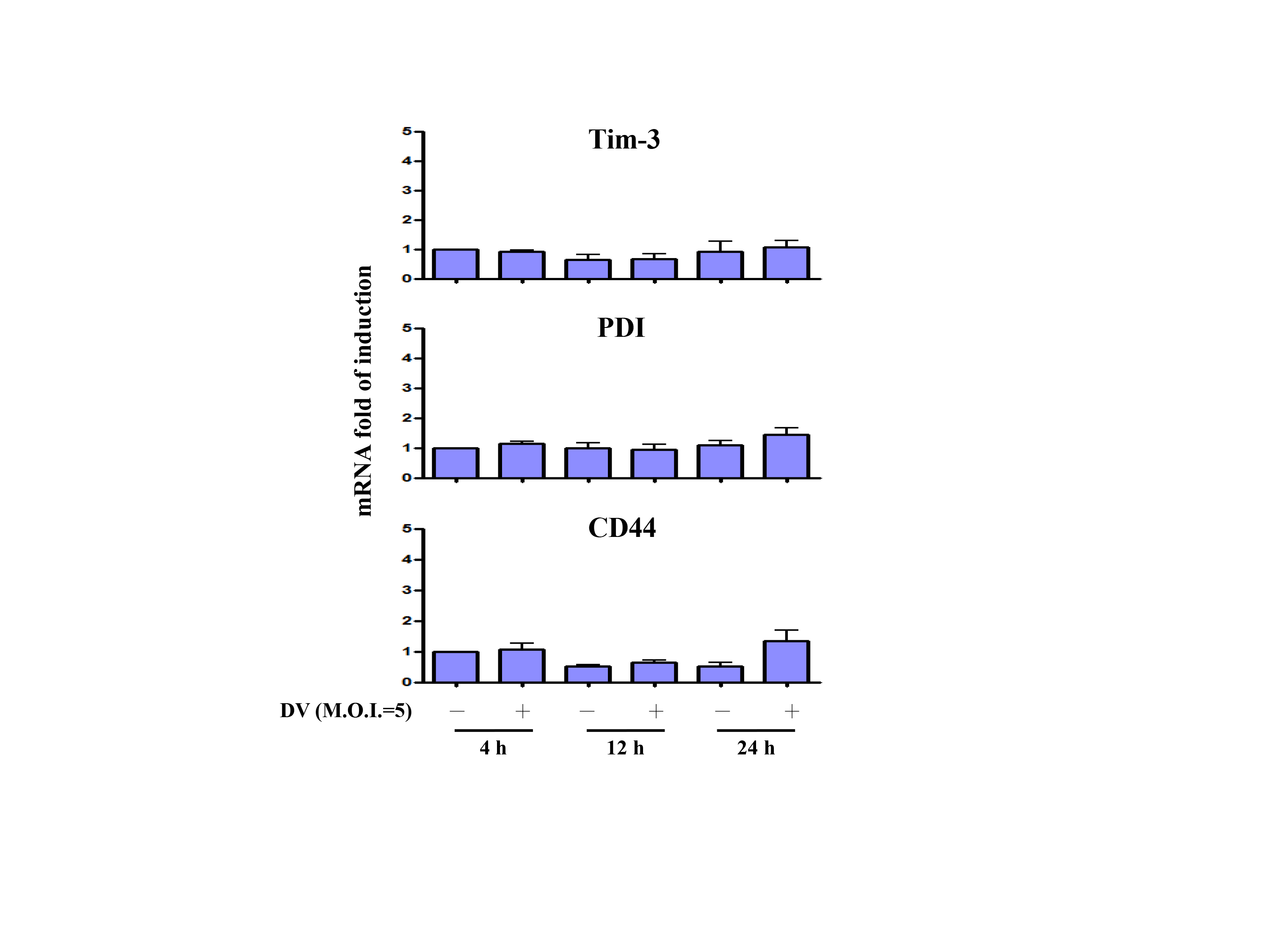
**

**Supplementary figure 1.** Expression of mRNA of Gal-9 receptors on DV-infected DCs. Human DCs were infected by mock or DV for 4 h, 12 h or 24 h. Expression of mRNA of Tim-3, PDI and CD44 genes were determined by quantitative RT/PCR. The data show the results pooled from at least 3 independent experiments using different donor cells.

**
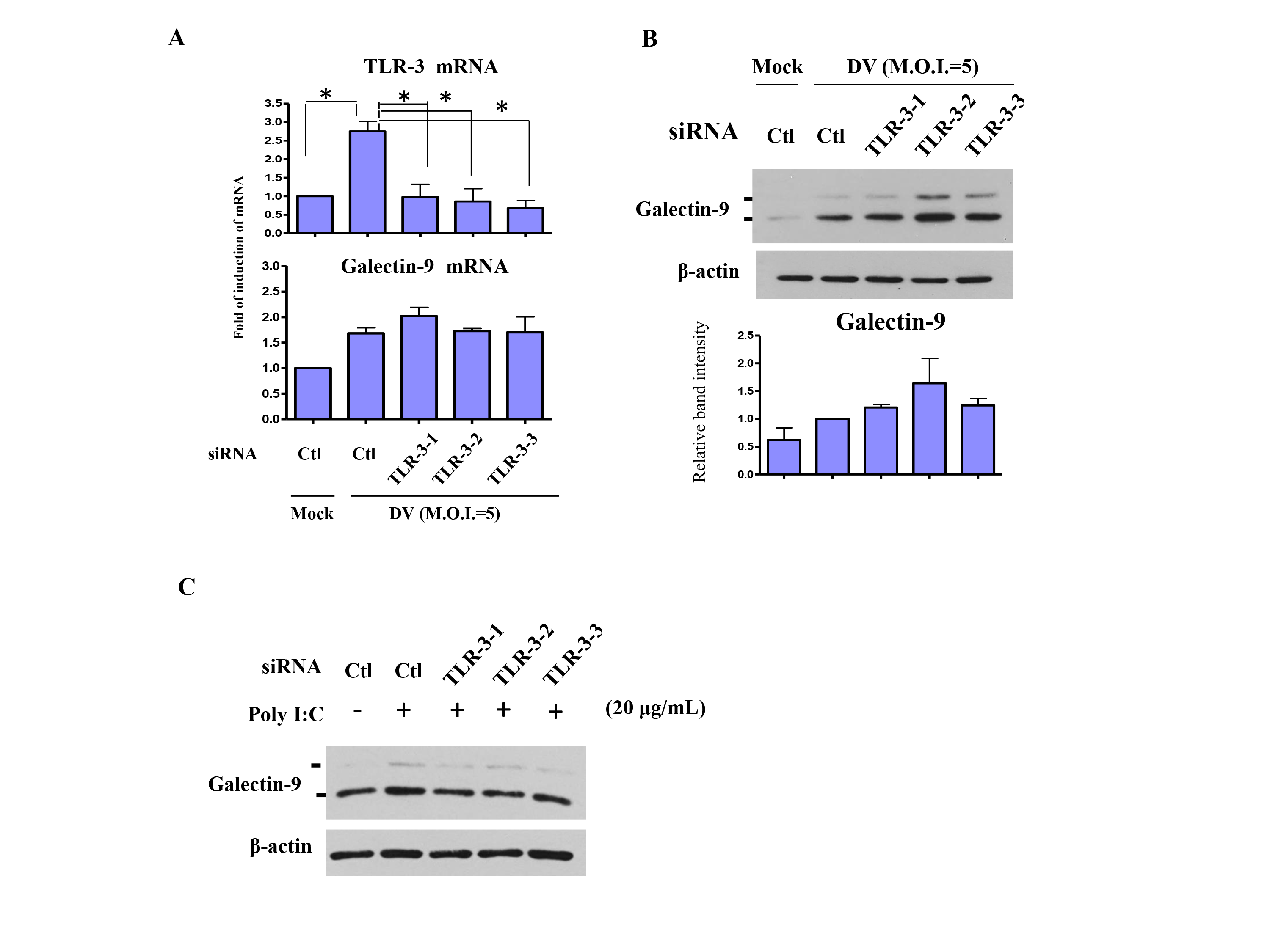
**

**Supplementary figure 2.** Knockdown of Toll-like receptor-3 (TLR-3) did not affect DV-induced Gal-9 expression. Human DCs were transfected with different siRNAs (Ctl, TLR-3-1, TLR-3-2 or TLR-3-3) for 24 h and then infected with mock or DV for 48 h. Expression of TLR-3 and Gal-9 mRNA was determined by quantitative RT/PCR (A). In (B), similar to (A), the levels of Gal-9 protein in the cell lysate were determined by Western blotting. The results pooled from at least 3 independent experiments examining different donor DCs were shown. In (C), human DCs were transfected with different siRNAs (Ctl, TLR-3-1, TLR-3-2 or TLR-3-3) for 24 h and then treated with or without 20 μg/ml Poly I:C for 48 h. The cell lysates were prepared and expression of Gal-9 and β-actin was determined by Western blotting. A representative result was shown.

**
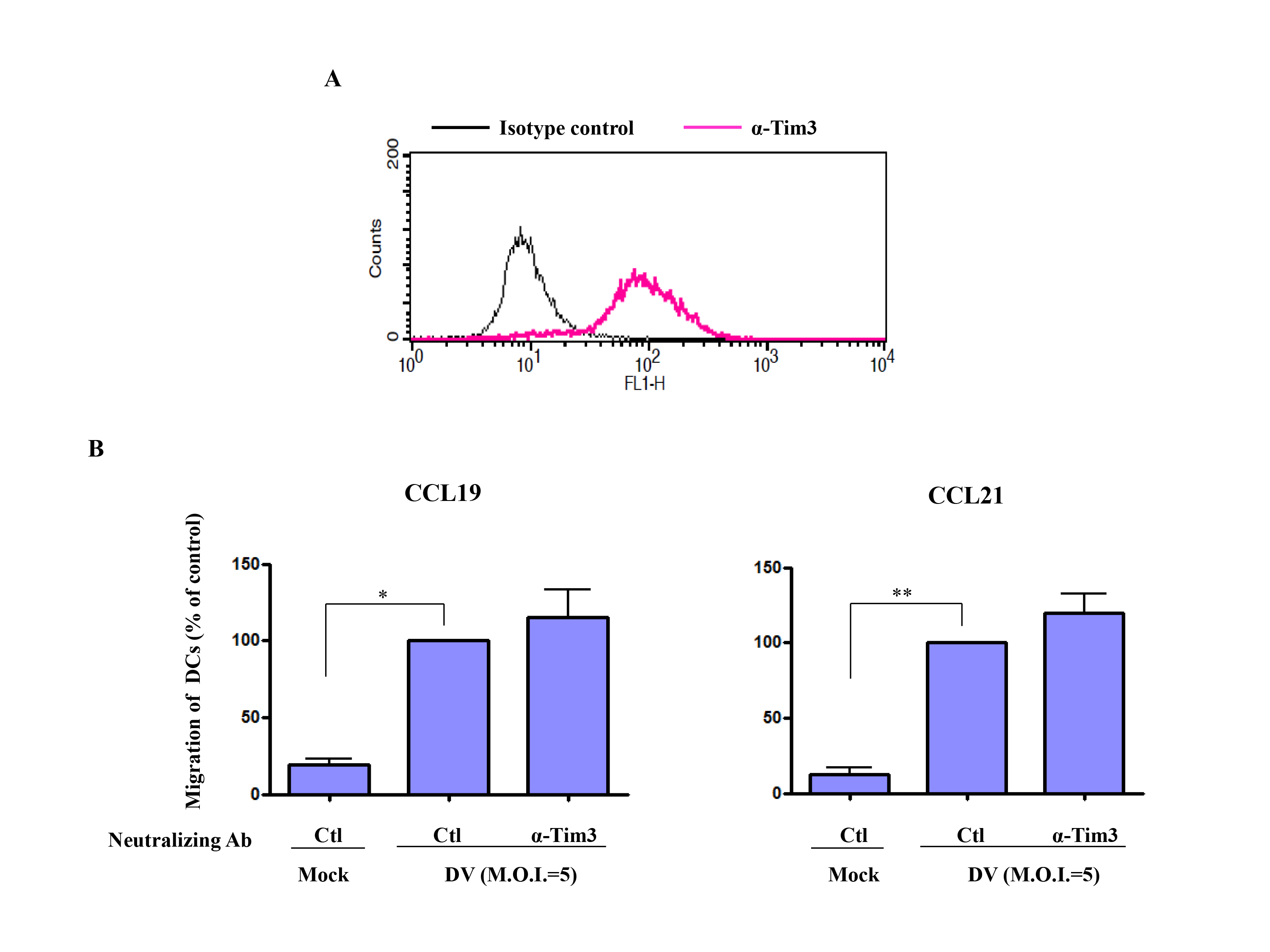
**

**Supplementary figure 3.** Blocking interaction between Tim-3 and Gal-9 did not affect DV-induced DC migration. (A) Flow cytometry analysis showed that the anti-Tim-3 neutralizing antibodies were able to recognize Tim-3 molecules on DCs. In (B), chemotaxis assays were performed as described in Fig.3 legend except the interaction between Gal-9 and Tim-3 was blocked by adding anti-Tim-3 neutralizing antibodies (10 μg/ml) or isotype-matched control antibodies for 2 h and then infected by mock or DV for 48 h. The data show the results pooled from at least 3 independent experiments using different donor cells. The analysis was performed by ANOVA, as described in the Materials and Methods. *P < 0.05. Ctl, control.


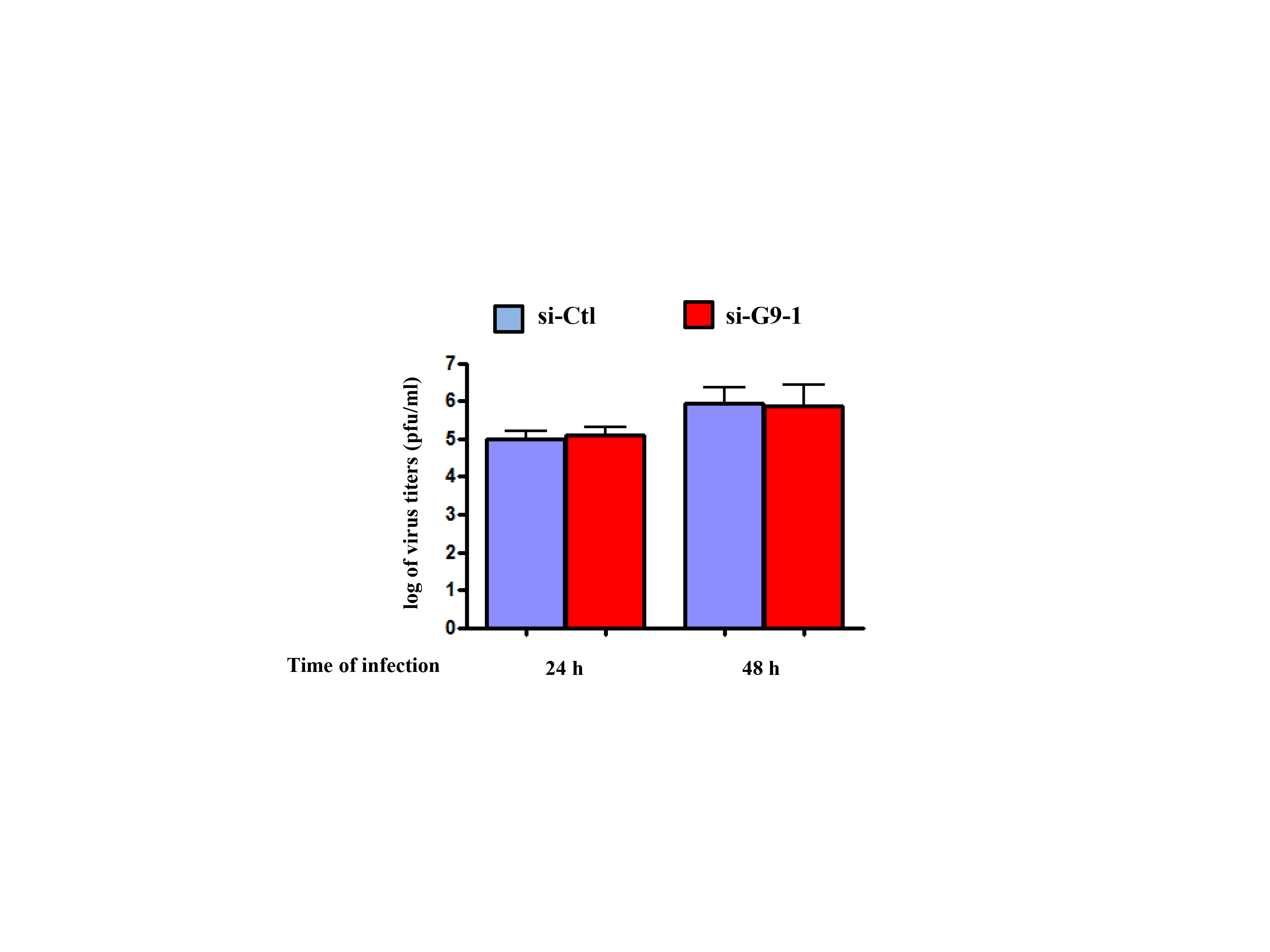


**Supplementary figure 4.** DV replication and production in infected DCs was independent of Gal-9. Human DCs transfected with control siRNA (si-Ctl) or Gal-9 siRNA (si-G9-1) for 24 h were infected by mock or DV at an MOI of 5 for an additional 24 h or 48 h. Supernatants were collected to determine virus titers by plaque assays. The data show results pooled from at least 3 independent experiments using different donor cells. Ctl, control.

**
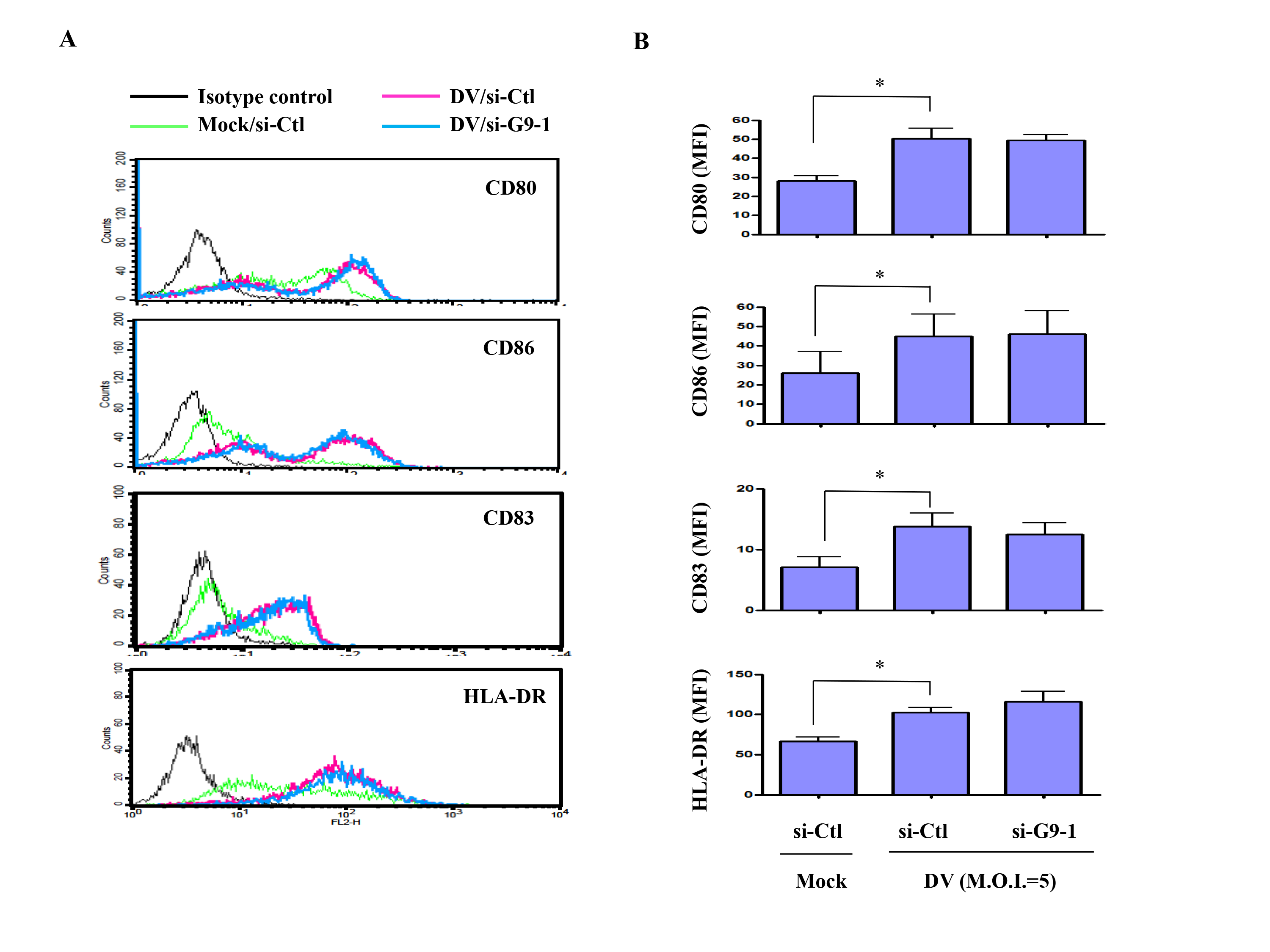
**

**Supplementary figure 5.** DV infection–induced expression of activation and maturation markers was not affected in Gal-9–deficient cells. Human DCs were transfected with control siRNA (si-Ctl) or Gal-9 siRNA (si-G9-1) for 24 h and then infected by mock or DV for an additional 48 h. Cells were collected for measurement of expression of CD80, CD86, CD83 and HLA-DR by flow cytometry (A). The data show results pooled from at least 3 independent experiments (B). The analysis was performed by ANOVA, as described in the Materials and Methods. *P < 0.05. Ctl, control; MFI, mean fluorescence intensity.

**
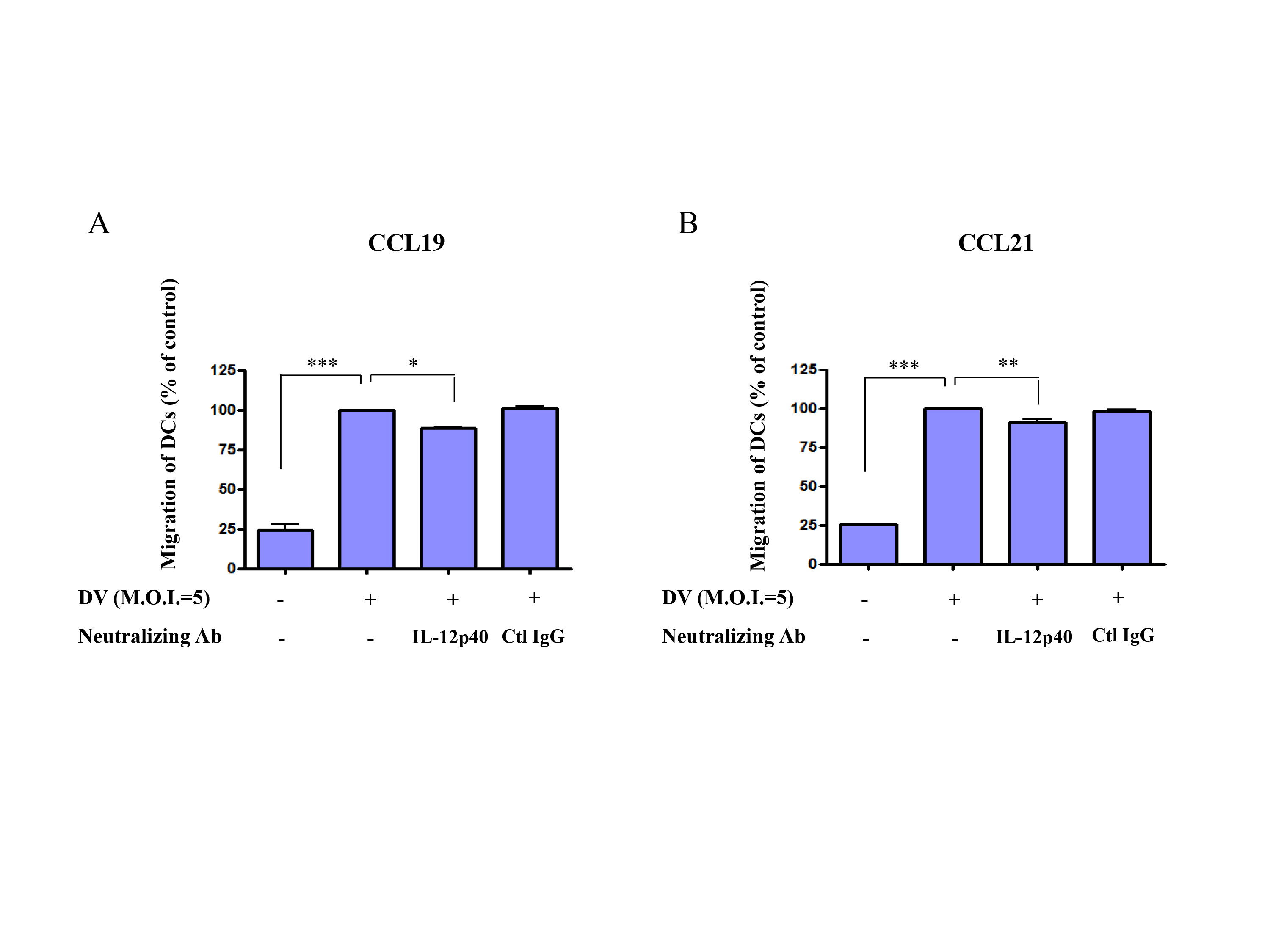
**

**Supplementary figure 6.** Neutralizing IL-12 significantly but only modestly suppressed DV-induced DC migration. Human DCs were pretreated with IL-12p40 (600 ng/ml) or goat IgG control (Ctl IgG) antibody for 2 h and then infected by mock or DV for 48 h. Cells were collected for measurement of chemotaxis activity by transwell assays using CCL19 (A) or CCL21 (B) as a chemoattractant. Data show representative results and analysis pooled from at least 3 independent experiments. The analysis was performed by ANOVA. *P < 0.05, **P < 0.01, ***P < 0.001.


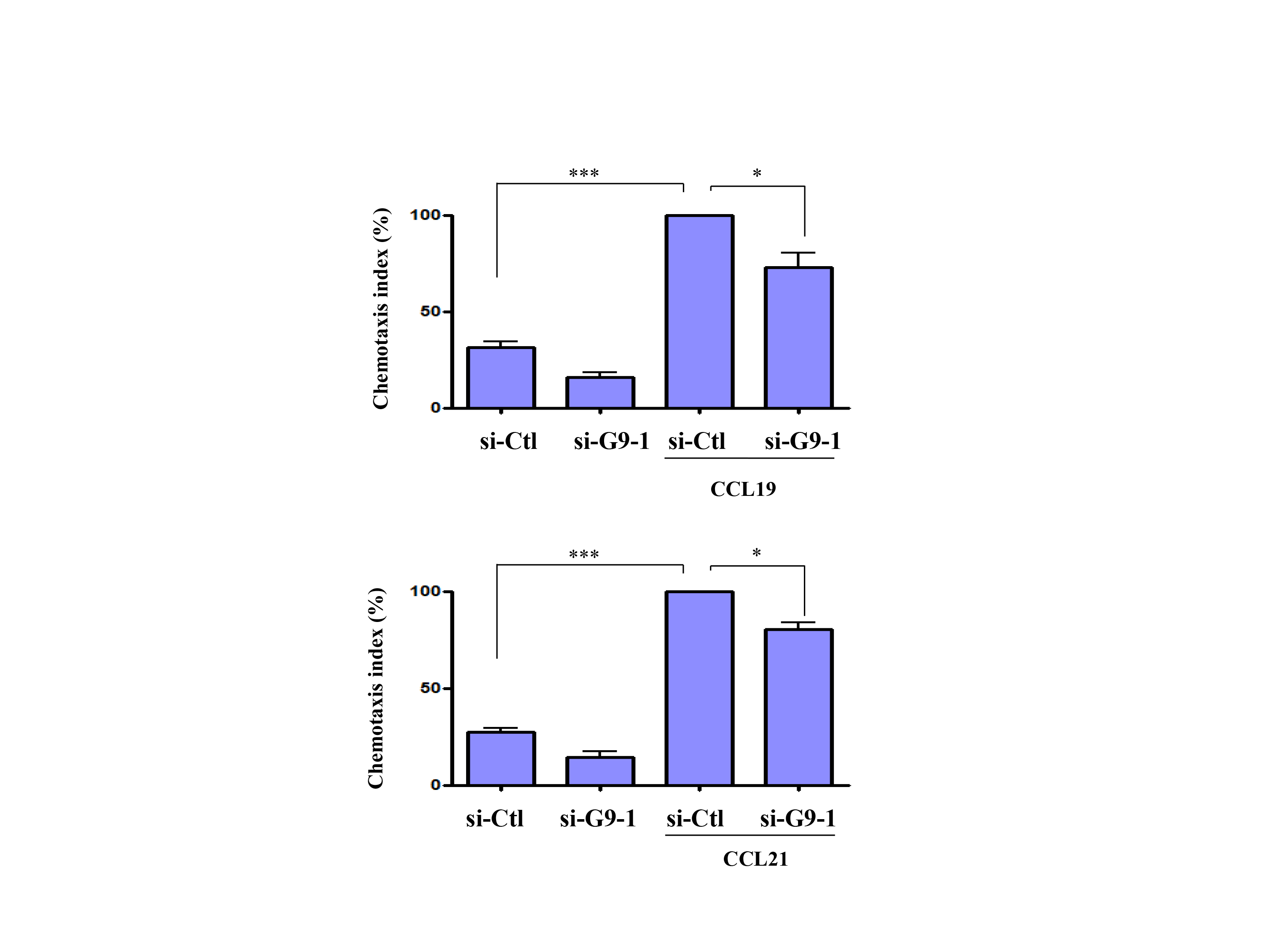


**Supplementary figure 7.** Chemotaxis of DV-infected DCs, with or without Gal-9 deficiency, toward the chemoattractants CCL19 and CCL21. Cells transfected with control siRNA (si-Ctl) or Gal-9 siRNA (si-G9-1) for 24 h were infected by DV for an additional 48 h. Cells were collected for measurement of chemotactic activity by transwell assays using CCL19 or CCL21 as a chemoattractant. The data show results pooled from at least 3 independent experiments using different donor cells. The analysis was performed by ANOVA. *P < 0.05, ***P < 0.001. Ctl, control.
